# Can Diabetes I and Early Blindness Be Prevented Using a Tylenol Combination Which Inhibits Oxidative and Nitrosative Stress?

**DOI:** 10.5402/2011/461928

**Published:** 2011-10-05

**Authors:** Knox Van Dyke, Erica Ghareeb, Robert Hoeldtke, Mark Van Dyke, Chris Van Dyke, David Van Thiel

**Affiliations:** ^1^Department of Biochemistry and Molecular Pharmacology, Robert C. Byrd Medical Center, West Virginia University Morgantown, WV 26506, USA; ^2^Department of Medicine, Robert C. Byrd Medical Center, West Virginia University Morgantown, WV 26506, USA; ^3^Center for Digestive Disorders at Rush Medical Center, Chicago, IL 60612, USA

## Abstract

Since oxidative/nitrosative stress cause diabetes, can we prevent this chemistry generating the disease? Streptozotocin causes diabetes by entering the pancreatic beta cell generating excessive nitric oxide which reacts with oxygen creating a toxin possibly peroxynitrite, dinitrogen trioxide, dinitrogen tetraoxide and so forth. The toxic compounds damage the DNA causing beta cell death. This prevents insulin synthesis, storage and release. By using antioxidant substances that destroy the nitric-oxide-based toxins (e.g., carboxy-PTIO (oxidizes nitric oxide), polyphenolic-quercetin and monophenolic acetaminophen (Tylenol)) which are oxidation and nitration targets can the diabetes I causing toxins in animals be destroyed? Will this tri-drug combination completely prevent the deleterious effects of diabetes namely poor blood glucose control and blindness from cataracts for the entire length of the experiment (one year). These disease reversal experiments were accomplished in rats where the streptozotocin-diabetic effects were completely thwarted. *In vitro* experiments were accomplished to provide the scientific basis for the experimental results in animals.

## 1. Introduction

 Type I diabetes, often called juvenile diabetes, results from the loss of active insulin-producing pancreatic-beta cells which causes an insulin deficit. Once insulin deficit occurs, glucose uptake and metabolism in a variety of tissues is decreased and hyperglycemia occurs. Excessive glucose in the blood and the loss of insulin produces vascular damage via glycosylation and activation of oxidative and nitrosative stress linked to all of the symptoms associated with diabetes, including peripheral and autonomic neuropathy, nephropathy, and retinopathy. Other pathology occurs such as atherosclerosis that can lead to eye, kidney, heart, and brain damage as well as peripheral vascular disease, infections, blindness, and gangrene with concomitant loss of limbs [1].

 The causes of pancreatic beta cell death can be genetic or caused by viral attack, which results in immune attack by *t*-lymphocytes and stimulation of macrophages. In addition, chemical attack that produces nitric oxide and superoxide that may form peroxynitrite could be involved from recent experiments done by a variety of investigators [2–5]. In fact, this same aforementioned chemical scenario is very likely to be the destructive chemistry seen in genetic and virally induced type I diabetes.

 If that is the case, an opportunity presents itself of preventing type I diabetes and its consequences regardless of the cause. If we could decrease the amount of nitric oxide and/or superoxide in the beta cell and prevent the action of peroxynitrite or other nitrating agents produced from nitric oxide by using harmless phenolic target molecules, we should be able to prevent or at least control the diabetes.

 Streptozotocin is an unusual molecule composed of a sugar on one side linked to a nitric oxide generator (nitrosourea) on the other. When a solution of the correct concentration is injected into a rodent, the animal develops juvenile or type I diabetes overnight. Therefore, if we simultaneously inject phenolic target molecules, for example, Tylenol, quercetin, and a nitric oxide metabolizer, for example, carboxy-PTIO with a molar proportional concentration of streptozotocin, the idea of prevention of type I diabetes and blindness could be tested. The following presentation will be accomplished to see if we can stop chemically-induced diabetes. Such a demonstration would point toward the production of excessive nitric oxide as being the initiator in streptozotocin diabetes and this raises the possibility it is involved with genetic-based diabetes as well. 

## 2. Methods

### 2.1. Drugs and Chemicals

**Figure 1:**
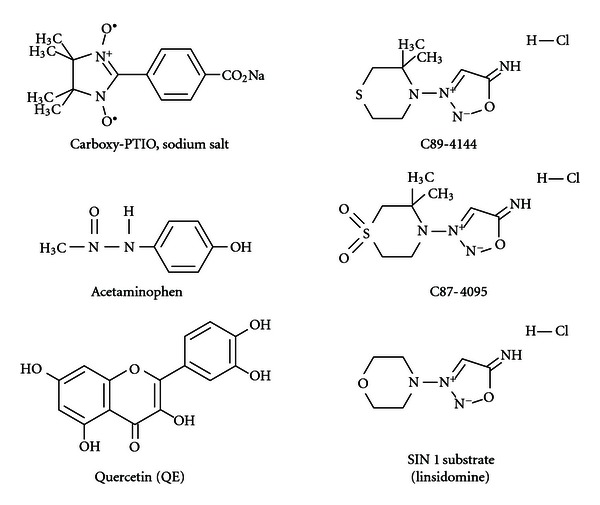
The chemical structures of carboxy-PTIO sodium salt, acetaminophen (Tylenol), quercetin, C89-4144, C87-4095, and SIN-1 (linsidomine).

Carboxy-PTIO was obtained from Cayman Chemical Company, Ann Arbor Mich, USA, 48108. Quercetin and streptozotocin, hepes, and phosphate-buffered saline (PBS) were purchased from Sigma/Aldrich Chemical Company, Saint Louis, Mo, USA, 63178. Tylenol (acetaminophen) was obtained from the McNeil Company, Fort Washington, Pa, USA. 19034. L-012 was purchased from Waco Chemical Company, Va, USA. SIN-1 and the other C-sydnoimines, C894095, C89144, were obtained from Casella AG, Frankfurt, Germany, and we thank Dr Karl Schönafinger.  Figure 1 shows the structures of carboxy PTIO, quercetin, Tylenol (acetaminophen), SIN-1, C894095, C89144, and streptozotocin.

### 2.2. Drug Treatment of Animals and Glucose Monitoring

Animals were treated humanely and housed according to the rules of the West Virginia University animal care committee. Sprague-Dawley male albino rats weighing about 300 grams were used in this study. A group of eight of these rats were injected subcutaneously with a combination of streptozotocin at 30 mg/Kg and 10 mg Tylenol (acetaminophen), 10 mg of carboxy-PTIO, and 5 mg quercetin dissolved in 0.1 M phosphate buffered saline (PBS). Six control rats were injected with 30 mg/Kg streptozotocin in (PBS) alone. The animals were examined 24 hours, and 6–12 months later for blood glucose levels (tail) using a Thera-Sense Flash glucose monitoring system purchased from Wal-Mart using the same glucose testing strips as used in measuring human-blood glucose.

Blood is withdrawn from the end of the barely snipped tail of a rat after first applying a local anesthetic salve (used in dentistry for surface anesthesia of the gums). The TheraSense glucose monitoring system is effective using a very small drop of blood applied to the edge of a glucose monitoring strip which was inserted into the instrument seconds before assay. The assay is calibrated with a known glucose solution prior to testing and the correct numbered test strip encoded into the device using buttons on the instrument. The test strip number must match the number on the display to get correct values.

### 2.3. Control Glucose Levels in Normal or Tridrug-Protected Diabetic Animals

Basically blood glucose from a control (noninjected animal) or a diabetic tridrug-protected animal varies from 130 mg/deciliter to about 70 mg/deciliter with the average glucose level about 100 mg/deciliter. The glucose level in a diabetic animal given streptozotocin at 30 mg/kg produces a reading of 300–350 mg/deciliter. The difference in blood glucose levels between a diabetic and nondiabetic animal is fourfold or more. We have produced this data in 10 different animals ranging from non-injected controls, tridrug protected streptozotocin animals or streptozotocin alone animals.

### 2.4. Luminescence Measurement to Demonstrate Antioxidant Activity

Quercetin, Tylenol, and carboxy-PTIO were tested for antioxidant activity using the reaction between L-012 (a luminol-like compound) and SIN-1 (a generator of peroxynitrite). This combination reacts together in a buffered solution to produce oxidation-based blue luminescence. If an antioxidant is present, it will shift or inhibit the kinetics of light production. Luminescence was performed in a Berthold 9505C 6 channel luminometer at 37°C. The original stock concentration of SIN-1 is 20 mg/5 mL PBS and frozen at −80°C to prevent breakdown. This is diluted 1 : 1000 prior to assay and kept in a bucket filled with ice to prevent premature degradation. It is always added last to initiate the luminescent reaction. The stock concentration of L-012 is 5 mg/50 mL PBS. Tylenol or quercetin were initially dissolved in a small volume of dimethyl sulfoxide (0.5 mL) and once dissolved they were diluted in PBS at various concentrations shown in the appropriate graphs from 1 millimolar starting concentration to less than micromolar quantities. The volume in each Berthold luminometry tube (3 mL tube) was constant at 500 microliters in all assays. All drugs and reactants were dissolved in PBS (pH 7.4). The reaction in each case was accomplished for 20 minutes at 37°C and integrated with KINB software provided by the Berthold Company, Wildbad, Germany.

### 2.5. Statistical Analysis

All important quantitative experiments in this work were accomplished four times or more and standard error of the mean applied to this data to demonstrate the variation from the mean. Then Student's *t*-test for significance was applied between like groups to determine confidence. Confidence level at (95%) was considered significant, but since the standard error was small in every case applied, a much higher significance occurred. In almost all of this data, no overlap on multiple runs happened. 

## 3. Results

**Figure 2:**
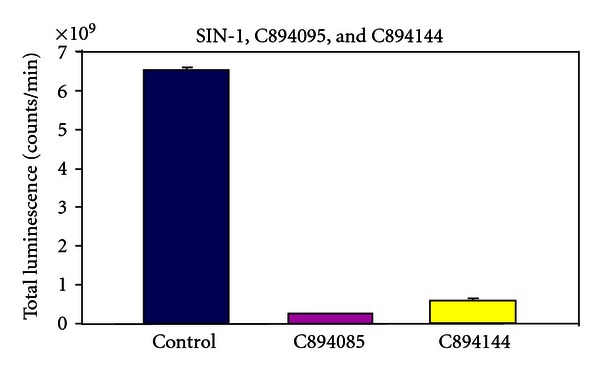
Total luminescent reactions of the different sydnoimines-SIN-1, C89-4095, C89-4144. Reaction conditions are detailed in Section 2. SIN-1 in equimolar concentration produces more luminescence than either of the other sydnoimines.

**Figure 3:**
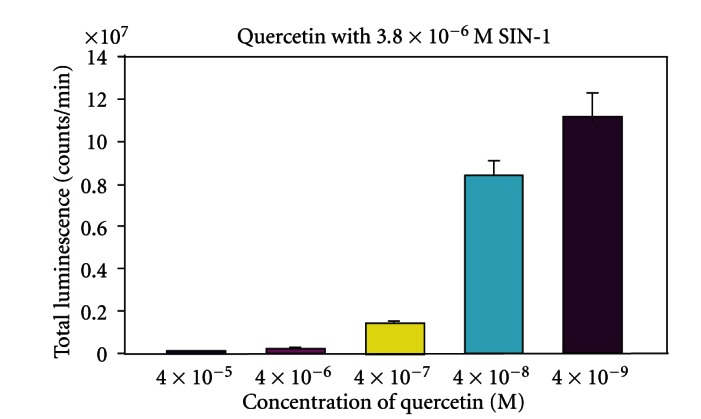
The effect of different molar concentrations of Tylenol on the luminescent reaction of L-012 and SIN-1. Reaction conditions are detailed in Section 2. The uninhibited control reaction gave luminescence of 2 × 10^8^ counts per minute.

**Figure 4:**
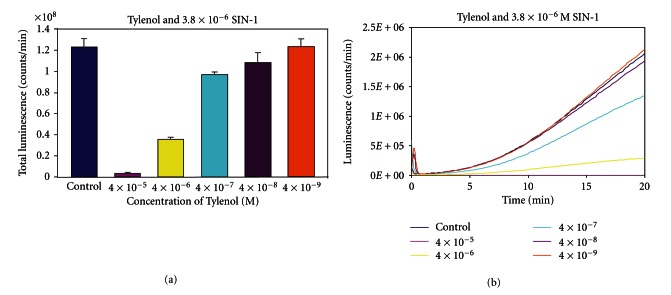
The inhibitory effect of quercetin in various doses against peroxynitrite(SIN-1) caused luminescent reaction of L-012. Reaction conditions are detailed in Section 2. The luminescence of L-012 and SIN-1 without quercetin was 2 × 10^8^ counts per minute.

**Figure 5:**
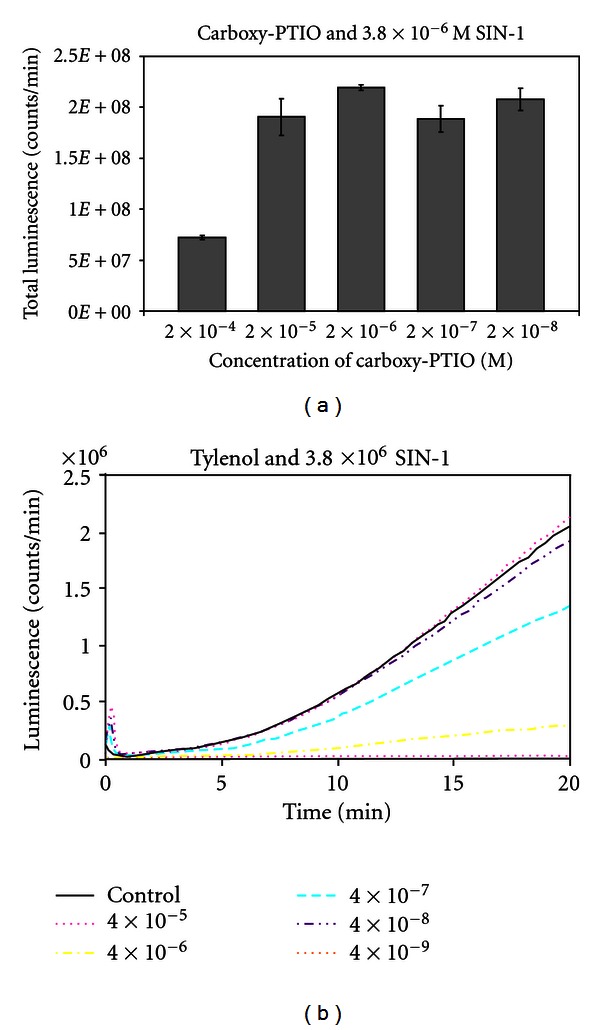
The effect of various doses of carboxy-PTIO on the luminescent reaction of L-012 and SIN-1. Reaction conditions are detailed in Section 2. The uninhibited reaction gave 2 × 10^8^ counts per minute.

**Figure 6:**
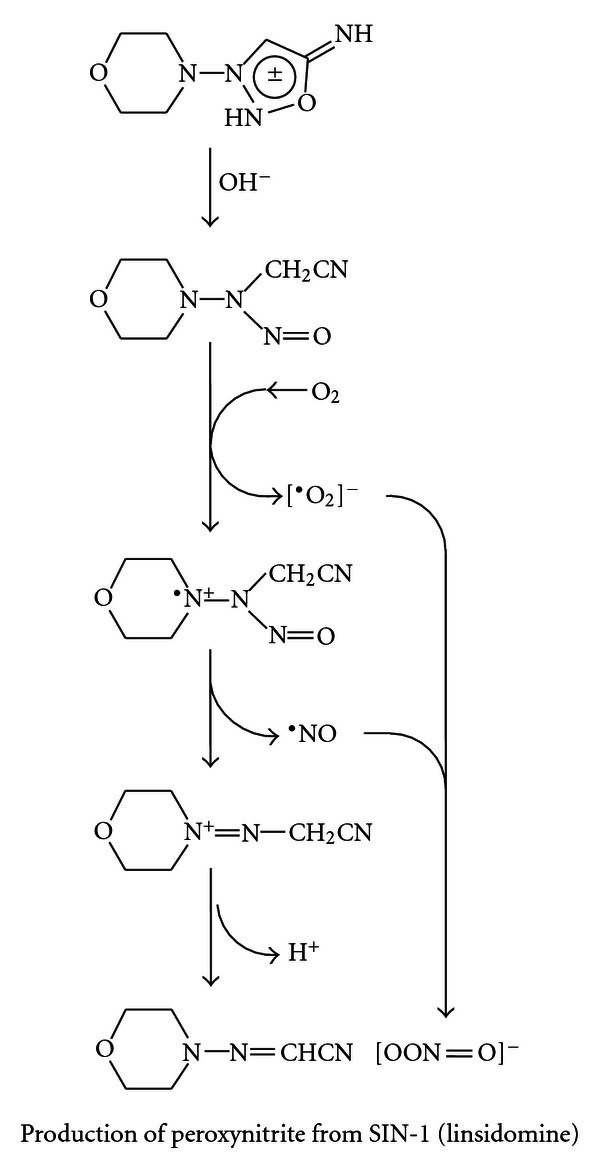
The degradative reaction sequence of SIN-1 in PBS to superoxide (^.^O_2_^−^) and nitric oxide (NO^∙^) free radicals which combine to produce peroxynitrite (OONO^−^). This is the compound which oxidizes L-012 to produce luminescence.

The luminometry system used in this paper depends on the oxidative attack of peroxynitrite (OONO^−^) generated by SIN-1, C894095, or C894144 (see Figure 2). Since SIN-1 produced the most luminescence or blue light with the best kinetics, it was chosen for the rest of the luminescence experiments. L-012 was chosen over the previous use of luminol [6], because it produces at least 100 fold or more intensity with equimolar concentration. In Figure 4, we see the dose response with Tylenol and SIN-1 in the presence of L-012 and complete inhibition of luminescence can be seen in micromolar amounts. In fact, significant inhibition of this oxidative system can be seen in doses 10-fold below the micromolar concentration of Tylenol. The second part of the figure displays the real-time kinetics of the reaction of various doses of the drug with peroxynitrite oxidation of L-012. In Figure 3, quercetin in micromolar concentration produces an inhibitory reaction of luminescence of the reaction of SIN-1 and L-012. In Figure 5, the effect of carboxy-PTIO was measured against the luminescent reaction of SIN-1 and L-012 and significant inhibition only occurred at 0.2 millimolar concentration. Multiple tenfold dilutions after that were essentially ineffective. Note that in Figure 6 is deployed the reaction scenario with SIN-1 and PBS buffer at pH = 7.4 to generate peroxynitrite. This peroxynitrite then reacts oxidatively with L-012 to produce blue luminescence. Therefore, various compounds that act as antioxidants like acetaminophen (a monophenol) and quercetin (a polyphenol) interfere with the production of luminescence. 

**Figure 7:**
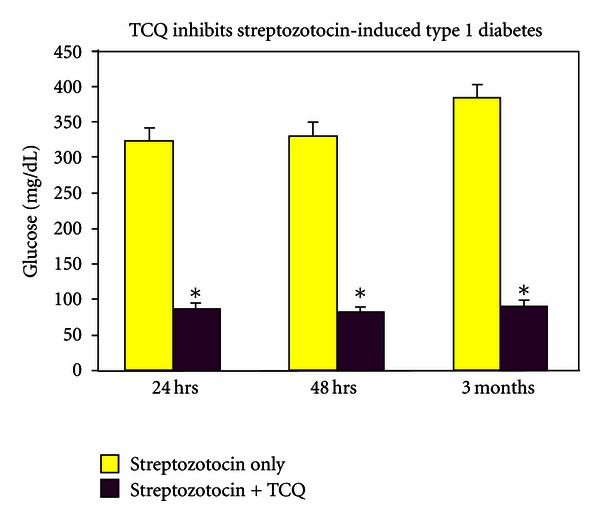
Blood glucose levels in rats treated with either streptozotocin (30 mg/kg) or streptozotocin (30 mg/kg), carboxy-PTIO (10 mg), quercetin 5 mg and Tylenol 5 mg. There were 6 animals in the first group and 8 animals in the second group. See Table 1 for individual glucose values from first and second group animals. See Section 2 for assay details.

The second major experiment was accomplished *in vivo* in male rats weighing about 300 grams and injected subcutaneously with either 30 mg/kg streptozotocin(S) or with S and 10 milligrams of Tylenol and carboxy-PTIO and 5 mg quercetin and with all substances mentioned dissolved in PBS. In intervals of twenty four hours, days, weeks, and many months thereafter, a small drop of blood was taken from the end of an anesthetized tail of each animal. We used 8 experimental animals injected with S and drugs and 6 control animals with S alone. The results of these experiments are displayed in Figure 7 and Table 1. The remarkable results show that S-injected animals were all clearly diabetic and that S and three drugs-injected animals were completely normal and protected from diabetes continuously (data not shown at all time points) and even one year later. This gives a statistical significance at a very high level with essentially a certain outcome that the three drugs have protected completely.

In Table 1 is depicted the blood glucose concentration of different individual rats in milligrams/deciliter units. Normal glucose levels are about 70–100 mg/deciliter. The diabetic rats display blood glucose values usually in the 300–400 mg/dL range (see Table 2). Once the animal is made diabetic with streptozocin the change is permanent, since the beta cell of the pancreas dies. The triple drug treatment with Tylenol, Quercetin, and Carboxy-PTIO given simultaneously with streptozotocin keeps the blood glucose of those rats in the normal range permanently possibly by protecting the beta cell of the pancreas from death.

**Figure 8:**
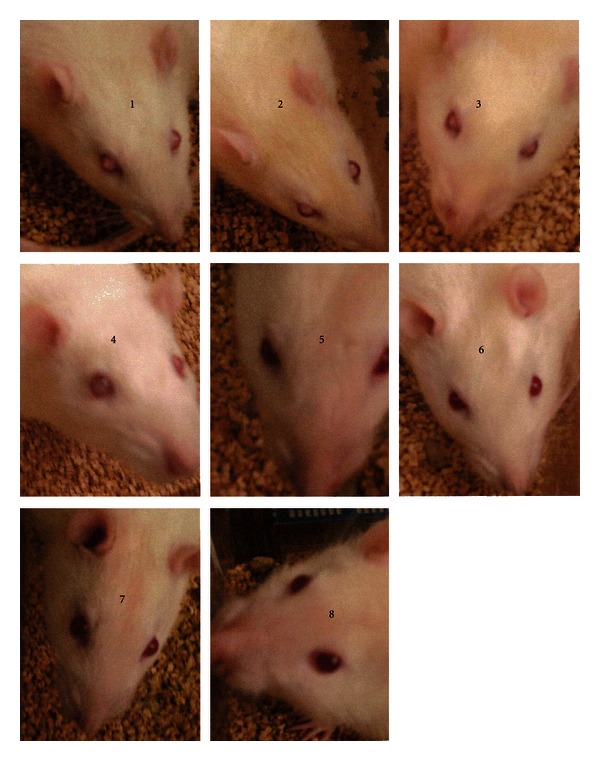
Rats Eyes. Animals were injected with 30 mg/kg streptozotocin alone (diabetic controls animals 1–4) or 30 mg streptozotocin and the three compounds carboxy PTIO, Tylenol-acetaminophen, and quercetin (animals 5–8). Note the animals with the three compounds did not develop eye clouding—an early step to blindness. These are animals 5–8. The animals 1–4 were injected with streptozotocin only have cloudy eyes.

In addition, injection with streptozotocin and the drug combination completely inhibited cloudiness in the eyes of these animals at all time intervals after 90 days. In animals injected with streptozotocin only, eyes from all animals became cloudy (see Figure 8).

## 4. Discussion

 The objective of this study was to determine the basic causative agent in type-one or juvenile diabetes. We chose a streptozotocin rat model, since it has been intensively studied and that it may represent the actual chemistry in the genetic and viral models of type-one diabetes. Further, there may be a linkage to adult or maturity onset diabetes as well. How could we stop the chemistry of streptozotocin from causing diabetes? To answer this, we would have to know how streptozotocin kills the beta cells of the pancreas causing the loss of insulin. A variety of studies have indicated that one end of the molecule is a sugar-like compound-deoxyglucose and that the other end is a nitric-oxide donating compound, an N methyl-nitrosourea moiety, where the N is part of a urea molecule linked to the deoxyglucose [2]. This compound generates nitric oxide (NO^∙^) inside a pancreatic beta cell in which it probably links to a form of oxygen eg superoxide (^.^O_2_^−^) from mitochondria and produces peroxynitrite (OONO-) or other NO-toxins. Since this is a highly energetic cell with low antioxidant protection, it is vulnerable to the oxidation and nitration properties of OONO^−^ or NO-toxic compounds which kills the beta cell, and thus prevents insulin's synthesis, storage, and secretion from this organ is lost [7]. It is possible that other molecules that are highly toxic could be made from superoxide and nitric oxide, for example, N_2_O_4_ or  NO_2_^∙^. Therefore, if we could reverse-engineer the chemistry of this beta cell killing system, maybe we could prevent this disease, diabetes. What type of chemicals could we use that would be safe and effective without major side effects? 

 At least in the short term, a compound like carboxy-PTIO that is a nitronyl nitroxide that converts NO^.^ to NO_2_^−^ might be helpful. According to a study by Durán-Reyes et al., carboxy-PTIO had some protective effect against the STZ-induced diabetes. Treatment of the STZ-induced diabetic rats with carboxy-PTIO (c-PTIO) after 24 hours returned insulin levels to more normal levels in 60% of the treated animals [2]. Furthermore, by combining c-PTIO with certain mono- and polyphenols (useful as target molecules for oxidation or nitration from peroxynitrite or other toxic nitrogen oxides) might help destroy the toxicity of these compounds. Drugs like Tylenol and/or natural polyphenolic compounds like quercetin might even synergize when given simultaneously. 

 We chose SIN-1 over the C type sydnoimines C 894095 or C 894144, since its reaction with L-012 was swift and vigorous even in small amounts (see Figure 2). We chose L-012 over luminol, since it produces blue light at equimolar doses about 100 times more than that of luminol. Tylenol (acetaminophen) exerts vigorous inhibition of luminescence at a concentration below micromolar level (see Figure 4). This drug level is certainly achieved in the blood [8] without significant toxicity. It becomes a most important candidate to help prevent damage in diabetes. Secondly, quercetin is a polyphenol that is found naturally in food sources, for example, fruits and vegetables, and it displayed major inhibition of peroxynitrite-based luminescence which was well below micromolar quantities clearly and clearly achievable in the blood (see Figure 3). Carboxy-PTIO did not display inhibition of luminescence at low concentrations. However, inhibition was seen only at the highest concentration 0.2 millimolar (see Figure 5), and this may be due to color quenching (blue drug) at this dose rather than actual inhibition. Since SIN-1 decomposes into (NO^.^) and (^.^O_2_^−^), the carboxy-PTIO ought to be very inhibitory at a variety of doses. The fact that it is not may be due to the possibility (NO^.^) is not completely released from SIN-1 until it reacts with its own superoxide. If that was the case, carboxy-PTIO may be ineffective with this type of reaction but not with the toxicity caused by excessive nitric oxide inside the beta cell of the pancreas, thereby protecting the major insulin supply. Carboxy-PTIO may react slowly with nitric oxide in a kinetic sense, but we know that superoxide and nitric oxide from the SIN-1 react at diffusional speed (10-9 sec) to form peroxynitrite before c-PTIO can react with nitric oxide.

 Therefore, a likely scenario might be to try carboxy-PTIO, Tylenol and quercetin together, since others have indicated that carboxy-PTIO is partially effective against streptozotocin diabetes [2]. Therefore, a combination of substances that can act as targets of nitration and a nitric oxide detoxifier inside the beta cell might be an effective combination against streptozotocin(S) diabetes, since (S) creates an excess of nitric oxide and likely an NO-based toxin inside the beta cell causing diabetic destruction and type one diabetes. The effect of this triple treatment on streptozotocin-based diabetes can be seen in Figure 7. In all six of the streptozotocin-injected animals, diabetes was seen in 24 and 48 hours and all days and months thereafter. In all eight of the Tylenol-, quercetin-, carboxy-PTIO-, and streptozotocin-injected animals, no diabetes was seen in any animal so injected 24 hours later or any day, month or a year (not shown) thereafter. From the literature, we have seen that this combination has not been published previously to completely thwart diabetes I caused by streptozotocin. It gives hope that this or a similar basic strategy might be helpful in human diabetes type I.

 In addition, after animals had been treated for 3 months, the streptozotocin-only injected animals developed severe clouding of the eyes (cataracts) that is a common symptom of blindness. Our streptozotocin animals treated with carboxy-PTIO, Tylenol, and quercetin completely prevented this as well as keeping the blood glucose at normal levels for multiple months. Clearly, these animals are not diabetic and display none of the signs of diabetes such as clouding of the eyes and high blood sugar levels (see Figure 8).

 A basic problem in human diabetes I is how and when can we recognize when diabetes is beginning. Diabetes is not generally recognizable in humans until 80%–90% of the Beta cells are dead. Recently, we have shown that antioxidant protection via blood urate is 25% diminished from early diagnosis—each and every year in early type I diabetic children. This type I diabetic oxidative and nitrosative stress was recognized in every patient tested (more than 30 children) [9]. Surely, the oxidative and nitrosative stress continues in the ongoing diabetic process causing depletion of the major antioxidant in the blood, namely, urate.

## 5. Conclusions

It is clear that previous approaches of controlling blood sugar levels or insulin levels has not been very effective, since the diabetic disease process continues. This is because the basis of the disease process, oxidative/nitrosative stress, has never been addressed. The data in this paper demonstrates that inhibiting oxidative and nitrosative stress early in the disease process can prevent the disease. We are currently working on a new noninvasive method to measure early oxidative/nitrosative stress in disease states. This will allow earlier interdiction into the actual disease state. We hope that this paper will inspire others to try different methods to prevent the chemical basis of diabetes. Banting in his Nobel Prize speech in 1922 mentioned that, “insulin is not the cure for diabetes.” Since diabetes is a primary cause of blindness and amputations, kidney disease, heart problems, and strokes, a method to thwart the chemical basis of diabetes would be particularly timely and relevant.

## Figures and Tables

**Table 1:** Rats treated simultaneously with streptozotocin and a Carboxy-PITO, Quercetin, and Tylenol mixture.

	24 h following treatment	48 h following treatment	3 months following treatment
Rat 1	92	84	85
Rat 2	71	64	55
Rat 3	83	83	ND
Rat 4	131	104	102
Rat 5	96	105	112
Rat 6	63	71	ND
Rat 7	79	75	ND
Rat 8	67	70	ND

**Table 2:** Streptozotocin-treated rats.

	24 h following treatment	48 h following treatment	3 months following treatment
Rat 1	332	345	342
Rat 2	364	367	437
Rat 3	238	242	ND
Rat 4	329	338	358
Rat 5	316	323	ND
Rat 6	369	360	392

## References

[1] Cotran RS (1999). *Robbins-Pathologic Basis of Disease*.

[2] Durán-Reyes G, Pascoe-Lira D, Vilar-Rojas C (2004). Diabetogenic effect of STZ diminishes with the loss of nitric oxide: role of ultraviolet light and carboxy-PTIO. *Pharmacology*.

[3] Turk J, Corbett JA, Ramanadham S, Bohrer A, McDaniel ML (1993). Biochemical evidence for nitric oxide formation from streptozotocin in isolated pancreatic islets. *Biochemical and Biophysical Research Communications*.

[4] Kwon A, Sakuri H (1994). Nitric oxide generation from streptozotocin. *FASEB Journal*.

[5] Tsuji A, Sakurai H (1998). Generation of nitric oxide from streptozotocin (STZ) in the presence of Copper(II) plus ascorbate: implication for the development of STZ-induced diabetes. *Biochemical and Biophysical Research Communications*.

[6] Van Dyke K, Sacks M, Qazi N (1998). A new screening method to detect water-soluble antioxidants: acetaminophen (Tylenol) and other phenols react as antioxidants and destroy peroxynitrite-based luminol-dependent chemiluminescence. *Journal of Bioluminescence and Chemiluminescence*.

[7] Spinas GA (1999). The dual role of nitric oxide in islet *β*-cells. *News in Physiological Sciences*.

[8] Prescott LF (1996). *Paracetamol (Acetaminophen): A Critical Bibliographic Review*.

[9] Van Dyke K, Jabbour N, Hoeldtke R, Van Dyke C, Van Dyke M (2010). Oxidative/nitrosative stresses trigger type i diabetes: preventable in streptozotocin rats and detectable in human disease. *Annals of the New York Academy of Sciences*.

